# A three-term conjugate gradient method with a new descent structure for unconstrained optimization and low-carbon supply chain optimization

**DOI:** 10.1371/journal.pone.0356498

**Published:** 2026-08-27

**Authors:** Maulana Malik, Sulaiman Mohammed Ibrahim, Dian Lestari, Fevi Novkaniza, Sindy Devila, Fida Fathiyah Addini, Gladwin Gunawan

**Affiliations:** 1 Department of Mathematics, Faculty of Mathematics and Natural Sciences, Universitas Indonesia, Depok, Indonesia; 2 Advanced Risk, Actuarial, and Financial Analytics Laboratory (ARAFA Lab), Universitas Indonesia, Depok, Indonesia; 3 College of Applied and Health Sciences, A’Sharqiyah University, Ibra, Sultanate of Oman; 4 Faculty of Education and Arts, Sohar University, Sohar, Oman; SRM University AP - Amaravati: SRM University AP, INDIA

## Abstract

Optimization methods play a vital role in solving large-scale problems across engineering and environmental fields. Among them, the conjugate gradient (CG) method is popular for its computational efficiency, and recent developments—such as the three-term CG variant—have shown improved convergence and numerical stability. This paper proposes an improved three-term CG method for unconstrained optimization. Inspired by the Three-Term Zheng-Huang-Shi (ZHS) CG parameterization and Three-Term Rivaie-Mustafa-Ismail-Leong (TTRMIL), this method is specifically designed to improve the computational performance of CG methods. In the proposed method, sufficient descent conditions and global convergence properties for general functions are mathematically proven under assumption and criteria from the strong Wolfe line search. Numerical experiments conducted on several unconstrained optimization problems highlight the superiority of the new method over certain CG methods with similar characteristics. In real-world applications, the proposed method is extended to address the challenge of reducing carbon emissions for sustainability, particularly within the Low Carbon Supply Chain (LCSC) optimization problem.

## 1 Introduction

Unconstrained optimization models arise frequently in applied mathematics [1–3], engineering [2,4], and machine learning [5–8]. These models are often expressed in the form


$$\begin{array}{c} \min_{x\in {\mathbb{R}}^{n}}f(x), \end{array}$$ (1)

where $f:{\mathbb{R}}^{n}\to \mathbb{R}$ is a smooth function whose gradient is given as g(x)=∇f(x) [1,9]. Problems of the form (1) are often solved using iterative methods, especially when the dimension of *n* is large, since direct methods are computationally infeasible [10].

Among the iterative techniques available in the literature, the nonlinear CG method has been widely studied due to its simplicity, efficiency in large-scale minimization problems, and low storage requirement [9]. The standard CG iterative scheme updates its sequence of iterative points using


$$\begin{array}{c} x_{k+1}=x_{k}+\alpha_{k}d_{k}, \end{array}$$ (2)

where $x_{k}\in {\mathbb{R}}^{n}$ denotes the current iterate, $x_{k+1}\in {\mathbb{R}}^{n}$ is the next iterate, $d_{k}\in {\mathbb{R}}^{n}$ is the search direction in iteration *k*, and $\alpha_{k}>0$ is the step size obtained by a suitable line search procedure, which could be an exact or inexact line search. The prominent line search strategy employed in literature is the Strong Wolfe technique that requires $\alpha_{k}$ to satisfy


$$\begin{array}{cc} f(x_{k}+\alpha_{k}d_{k}) & \leq f(x_{k})+\psi \alpha_{k}g_{k}^{T}d_{k}, \end{array}$$ (3)

$$\begin{array}{cc} \left|g_{k+1}^{T}d_{k}\right| & \leq -\sigma g_{k}^{T}d_{k}, \end{array}$$ (4)

where 0<ψ<σ<1. The parameters ψ and σ in (3)–(4) control the balance between sufficient decrease and curvature condition. Particularly, ψ ensures a sufficient decrease in the function value and is usually chosen small. On the other hand, σ enforces the curvature condition and is generally larger. The inequality 0<ψ<σ<1 guarantees descent, practical step sizes, and global convergence [1,9,10].

Another important feature from (2) is the search direction $d_{k}$ which is computed via the following formula:


$$\begin{array}{c} d_{k}=\left\{ \begin{array}{cc} -g_{k}, & k=0,\\ -g_{k}+\beta_{k}d_{k-1}, & k\geq 1, \end{array}\right. \end{array}$$ (5)

with the scalar $\beta_{k}$ representing the CG parameter that distinguishes different CG variants. Some of the well-known CG coefficients include: Hestenes–Stiefel [11], Polak–Ribière–Polyak [12,13], Fletcher–Reeves [14], and Dai–Yuan [15] with formulas as follows


$$\begin{array}{c} \beta_{k}^{HS}=\frac{g_{k}^{T}y_{k-1}}{d_{k-1}^{T}y_{k-1}},\;\beta_{k}^{PRP}=\frac{g_{k+1}^{T}y_{k}}{||g_{k}|{|}^{2}},\;\beta_{k}^{FR}=\frac{||g_{k+1}|{|}^{2}}{||g_{k}|{|}^{2}},\;\beta_{k}^{DY}=\frac{g_{k+1}^{T}g_{k+1}}{d_{k-1}^{T}y_{k-1}}. \end{array}$$ (6)

where $y_{k-1}=g_{k}-g_{k-1}.$ These earlier versions of CG gradient formulas (see; (6)) of possesses distinct numerical and theoretical characteristics. As an illustration, the HS parameter is characterized by a high degree of practical performance and is able to produce effective search directions, especially when the line search strategies are used, but the HS method can be unstable when the numerator is small (the denominator $d_{k-1}^{T}y_{k-1}$), which can compromise the global convergence. The PRP procedure can frequently be rapidly converged in practice and numerically efficient, though, unless precautions are taken, its formula does not necessarily guarantee descent, and the procedure can be subject to cycling. Conversely, the FR parameter has a simpler structure and high global convergence rates when the Wolfe conditions are met, but it can be overly conservative and converge sluggishly in ill-posed problems. In addition, the DY algorithm is better at global convergence and provides descent under weaker conditions, but its empirical performance can be inferior to that of HS or PRP on large-scale problems.

It is on the basis of these strengths and weaknesses that Hager and Zhang [16] developed a new CG parameter based on the HS structure and included a correction term to enforce descent and improve global convergence. Their procedure, which is as follows, modifies the HS formula by introducing a well-designed correction that ensures loss of descent is not lost and has numerical stability, thus synthesizing the high practical efficacy of HS with better theoretical guarantees


$$\begin{array}{c} \beta_{k}^{HZ}=\frac{1}{d_{k-1}^{T}y_{k-1}}\left(y_{k-1}^{T}g_{k}-2\frac{\|y_{k-1}{\|}^{2}}{d_{k-1}^{T}y_{k-1}}\;d_{k-1}^{T}g_{k}\right). \end{array}$$ (7)

The additional term


$$\begin{array}{c} -\;2\frac{\|y_{k-1}{\|}^{2}}{(d_{k-1}^{T}y_{k-1}{)}^{2}}\;d_{k-1}^{T}g_{k} \end{array}$$

which differentiates the HZ method from the classical HS parameter in (6) and ensures that the direction of search satisfies a sufficient descent condition irrespective of the line search procedure. The authors proved that their new method achieves global convergence under the Wolfe conditions, and further demonstrated the strong numerical efficiency on large-scale unconstrained optimization problems.

The Hager–Zhang modification represents an important enhancement in the category of two-term nonlinear CG algorithms. By incorporating the additional term into the HS algorithm, it successfully strengthens the descent condition and improves global convergence under mild inexact line search conditions. Nevertheless, the HZ scheme, like other classical variants, retains the inherent two-term structure


$$\begin{array}{c} d_{k}=-g_{k}+\beta_{k}d_{k-1}, \end{array}$$

which may still experience loss of conjugacy and numerical instability in ill-conditioned or highly nonlinear large-scale problems. These structural limitations are not solely due to the choice of the parameter $\beta_{k}$, but are also linked to the restricted memory of the two-term recurrence.

To further improve stability and preserve conjugacy more effectively, researchers introduced three-term conjugate gradient directions in which an additional correction component is incorporated. The general three-term structure is defined as


$$\begin{array}{c} d_{k}=-g_{k}+\beta_{k}d_{k-1}+\gamma_{k}d_{k-2},\;k\geq 2, \end{array}$$ (8)

where the scalar $\gamma_{k}$ denotes the three-term parameter [17–20]. Inclusion of the additional term $d_{k-2}$ not only enhances the numerical stability of the method but also helps to preserve the conjugacy more effectively [21,22]. As a result, three-term directions often provide improved performance and stronger results.

In the past few years, Zheng and Shi [23] have proposed a three-term CG method with a new search direction as follows:


$$\begin{array}{c} d_{k}=\left\{ \begin{array}{cc} -g_{k}, & k=0\\ -g_{k}+\beta_{k}d_{k-1}-\frac{g_{k}^{T}d_{k-1}}{\|g_{k-1}{\|}^{2}}y_{k-1}, & k\geq 1 \end{array}\right. \end{array}$$

where $\beta_{k}$ is defined as


$$\begin{array}{c} \beta_{k}^{ZHS}=\frac{g_{k}^{T}y_{k-1}}{\max\left\{\eta \|d_{k-1}\|\|y_{k-1}\|,d_{k-1}^{T}y_{k-1}\right\}}, \end{array}$$ (9)

for η>0, and $y_{k-1}=g_{k}-g_{k-1}$. The method is named the ZHS method. Under the Armijo line search, the ZHS method satisfies the global convergence properties and possesses the sufficient descent condition.

Recently, Liu et al. [24] proposed a new type of three-term CG method where the search direction is defined as follows:


$$\begin{array}{c} d_{k}=\left\{ \begin{array}{cc} -g_{k}, & k=0\\ -g_{k}+\beta_{k}d_{k-1}+\theta_{k}y_{k-1}, & k\geq 1 \end{array}\right. \end{array}$$ (10)

where


$$\begin{array}{c} \beta_{k}^{RMIL}=\frac{g_{k}^{T}y_{k-1}}{\|d_{k-1}{\|}^{2}}, \end{array}$$ (11)

and


$$\begin{array}{c} \theta_{k}=-\frac{g_{k}^{T}d_{k-1}}{\|d_{k-1}{\|}^{2}}. \end{array}$$ (12)

Researchers call this approach the TTRMIL method. The convergence analysis uses the Wolfe line search, and numerical experiments confirm that it effectively solves unconstrained optimization problems.

It is worth noting that the ZHS and TTRMIL methods possess complementary theoretical and numerical characteristics. The ZHS method introduces a safeguarded denominator through the max operator. This prevents the denominator from becoming excessively small, thereby improving numerical stability [23]. This safeguard plays an important role in ensuring the sufficient descent condition and global convergence, particularly when the gradients exhibit irregular behavior. However, the structure of the ZHS method does not fully exploit the flexibility of the additional third-term component to enhance curvature approximation.

On the other hand, the TTRMIL method incorporates an explicit third-term correction through the parameter $\theta_{k}$ [24], which enhances the ability of the search direction to capture second-order information implicitly [11,25]. This structure improves the quality of the search direction and often accelerates convergence in practice. Nevertheless, since the denominator of $\beta_{k}^{RMIL}$ and $\theta_{k}$ depends solely on $\|d_{k-1}{\|}^{2}$, the method may suffer from reduced robustness when the previous direction becomes nearly orthogonal to the gradient or when $\left\|d_{k-1}\right\|$ is small.

Therefore, by combining the safeguarded denominator mechanism of the ZHS method with the flexible three-term correction structure of the TTRMIL method, the resulting search direction benefits from both improved stability and enhanced curvature exploitation. Specifically, the safeguard from ZHS helps maintain numerical stability and descent properties even in challenging scenarios, while the three-term structure from TTRMIL captures curvature information more effectively, potentially accelerating convergence. This rationale underpins the design of the proposed method, which aims for both robustness and efficiency.

Motivated by these developments, we propose in this paper a new three-term CG direction, referred to as TTMZHS, that combines and extends ideas from ZHS and TTRMIL conjugate gradient methods. The proposed method is designed to:

1. Ensure the sufficient descent condition,
2. Achieve global convergence under the strong Wolfe line search,
3. Improve robustness and efficiency for large-scale optimization problems.

Beyond the theoretical results, we evaluate the proposed TTMZHS CG method on a large collection of benchmark test problems, demonstrating its superiority in terms of iterations, function evaluations, gradient evaluations, and CPU time compared to related methods. Finally, to highlight its real-world applicability, the method is applied to the LCSC optimization problem, which considers environmental and economic trade-offs simultaneously.

The rest of this paper is structured as follows. In Section 2, the study presented the derivation process of the new method and established its convergence properties under suitable conditions. Section 3 evaluates the numerical efficiency of the proposed method on a set of unconstrained optimization problems. Section 4 demonstrates the applicability of the new algorithm to the LCSC optimization problem. Lastly, Section 5 concludes the study with remarks and future research directions.

## 2 Formulation and global convergence

This section presents the TTMZHS CG method, a newly proposed three-term CG algorithm for solving the unconstrained optimization problem. The method is constructed to satisfy the sufficient descent condition and to achieve global convergence under the strong Wolfe line search.

### 2.1 Search direction formula of TTMZHS method

Motivated by the ZHS and TTRMIL CG methods, we propose a new three-term CG search direction by modifying both $\beta_{k}$ and $\theta_{k}$ in (10). Specifically, the parameter $\beta_{k}^{RMIL}$ is replaced by a safeguarded coefficient $\beta_{k}^{MZHS}$ defined as follows:


$$\begin{array}{c} \beta_{k}^{MZHS}=\frac{g_{k}^{T}y_{k-1}}{\max\left\{\eta \|d_{k-1}\|\|y_{k-1}\|,-g_{k}^{T}d_{k-1},\|g_{k-1}{\|}^{2}\right\}}. \end{array}$$ (13)

This modification enhances robustness by preventing instability from unfavorable geometric relationships between gradients and search directions. As a result, the proposed method delivers more reliable convergence, improves curvature-adaptation accuracy, and maintains consistent performance across various scenarios.

Similarly, the parameter $\theta_{k}^{MZHS}$ is defined as


$$\begin{array}{c} \theta_{k}^{MZHS}=\frac{-g_{k}^{T}d_{k-1}}{\max\left\{\eta \|d_{k-1}\|\|y_{k-1}\|,-g_{k}^{T}d_{k-1},\|g_{k-1}{\|}^{2}\right\}}, \end{array}$$ (14)

which uses the same safeguarded denominator to maintain consistency between the two parameters. This unified denominator structure helps preserve descent properties.

Hence, the proposed TTMZHS CG method generates the search direction as follows:


$$\begin{array}{c} d_{k}=\left\{ \begin{array}{cc} -g_{k}, & k=0\\ -g_{k}+\beta_{k}^{MZHS}d_{k-1}+\theta_{k}^{MZHS}y_{k-1}, & k\geq 1 \end{array}\right. \end{array}$$ (15)

The complete procedure of the proposed TTMZHS CG method is summarized in Algorithm 1.

**Algorithm 1 TTMZHS Conjugate Gradient Method**

**Require:** $x_{0}\in {\mathbb{R}}^{n}$, ϵ>0, $K_{\max}$, η>0, ψ∈(0,1), σ∈(ψ,1)

**Ensure:** Approximate solution $x_{k}$

1: k←0

2: $g_{0}\leftarrow \nabla f(x_{0})$, $d_{0}\leftarrow -g_{0}$

3: **while** $\|g_{k}\|>\epsilon$ **and** $k<K_{\max}$ **do**

4: Find $\alpha_{k}>0$ satisfying the strong Wolfe conditions (3) and (4).

5: $x_{k+1}\leftarrow x_{k}+\alpha_{k}d_{k}$

6: $g_{k+1}\leftarrow \nabla f(x_{k+1})$, $y_{k}\leftarrow g_{k+1}-g_{k}$

7: $M_{k}\leftarrow \max\{\eta \|d_{k}\|\|y_{k}\|,\;-g_{k+1}^{T}d_{k},\;\|g_{k}{\|}^{2}\}$

8: $\beta_{k+1}^{MZHS}\leftarrow \frac{g_{k+1}^{T}y_{k}}{M_{k}}$

9: $\theta_{k+1}^{MZHS}\leftarrow \frac{-g_{k+1}^{T}d_{k}}{M_{k}}$

10: $d_{k+1}\leftarrow -g_{k+1}+\beta_{k+1}^{MZHS}d_{k}+\theta_{k+1}^{MZHS}y_{k}$

11: k←k+1

12: **end while**

13: **return** $x_{k}$

### 2.2 Convergence analysis of the TTMZHS under strong wolfe line search

In this section, the descent condition and global convergence properties of the TTMZHS method are established under a strong Wolfe line search.

#### 2.2.1 Descent condition.

In this section, we establish the descent condition and global convergence properties of the TTMZHS CG method, specifically under a strong Wolfe line search—a procedure used in optimization that determines an appropriate step size by satisfying certain criteria for sufficient decrease and curvature.

The descent condition is fundamental in optimization because it ensures each iteration moves in a direction that reduces the objective function value. Specifically, a vector $d_{k}$ is a descent direction if


$$\begin{array}{c} g_{k}^{T}d_{k}<0, \end{array}$$ (16)

meaning the projection of the gradient onto $d_{k}$ is negative. Consequently, there exists a step size α>0 such that $f(x_{k}+\alpha_{k}d_{k})<f(x_{k})$, so the function value decreases along $d_{k}$ [26].

A direction $d_{k}$ is a sufficient descent direction if it not only satisfies the descent condition (16), but also ensures a large enough decrease in the objective function. This is often expressed as


$$\begin{array}{c} g_{k}^{T}d_{k}\leq -c\|g_{k}{\|}^{2}, \end{array}$$ (17)

for some constant *c* > 0. This guarantees that $d_{k}$ stays well aligned with the negative gradient. Such a condition helps ensure global convergence of optimization algorithms under certain line search strategies [27].

The following theorem will be used to illustrate that the proposed TTMZHS CG method satisfies the sufficient descent condition (17).

**Theorem 2.1.** *Suppose that the sequence* $\left\{x_{k}\right\}$ *is generated by Algorithm 1 where step size* $\alpha_{k}$ *is calculated by any line search, then the search direction (15) satisfies the sufficient descent condition with c = 1.*

*Proof.* For *k* = 0, from (15) we get $g_{0}^{T}d_{0}=-g_{0}^{T}g_{0}=-\|g_{0}{\|}^{2}$, so that (17) holds.

Next, for k≥1 and multiplying (15) by $g_{k}^{T}$, we have


$$\begin{array}{c} g_{k}^{T}d_{k}=-\|g_{k}{\|}^{2}+\beta_{k}^{MZHS}g_{k}^{T}d_{k-1}+\theta_{k}^{MZHS}g_{k}^{T}y_{k-1}. \end{array}$$ (18)

Substituting (13) and (14) into (18), we obtain


$$\begin{array}{cc} g_{k}^{T}d_{k} & =-\|g_{k}{\|}^{2}+\frac{g_{k}^{T}y_{k-1}}{\max\left\{\eta \|d_{k-1}\|\|y_{k-1}\|,-g_{k}^{T}d_{k-1},\|g_{k-1}{\|}^{2}\right\}}g_{k}^{T}d_{k-1}\\ & \;-\frac{g_{k}^{T}d_{k-1}}{\max\left\{\eta \|d_{k-1}\|\|y_{k-1}\|,-g_{k}^{T}d_{k-1},\|g_{k-1}{\|}^{2}\right\}}g_{k}^{T}y_{k-1}\\ & =-\|g_{k}{\|}^{2}. \end{array}$$

Therefore, from the above argument, the descent condition (17) holds with *c* = 1.□

In this study, we make the following assumption, which are fundamental for establishing the convergence properties of the proposed method.

**Assumption 2.2.** *1. The function f is bounded below on S_0_, and there exists a constant* ζ>0 *such that*


$$\begin{array}{c} \|g(x)\|\leq \zeta ,\;\forall x\in S_{0}. \end{array}$$ (19)

*2. The function f is continuously differentiable in an open neighborhood* $\Gamma ⊇S_{0}$*, and its gradient is Lipschitz continuous. That is, there exists a constant L > 0 such that*


$$\begin{array}{c} \|g(x)-g(y)\|\leq L\|x-y\|,\;\forall x,y\in \Gamma . \end{array}$$ (20)

The following lemma is fundamental for analyzing conjugate gradient methods. Zoutendijk originally introduced this condition under the Wolfe line search [28], and it also applies to exact, Armijo-Goldstein, and strong Wolfe strategies [29]. This lemma verifies the Zoutendijk condition under the strong Wolfe line search, reinforcing the global convergence of conjugate gradient algorithms. The proof follows that for the standard Wolfe condition [29], adapted here to the strong Wolfe line search for the TTMZHS CG method.

Before presenting the lemma on the Zoutendijk condition, we first establish a preliminary result that will be used in its proof.

**Lemma 2.3.** *If the step size* $\alpha_{k}$ *satisfies the strong Wolfe conditions (3) and (4), and Assumption 2.2 together with the sufficient descent condition (17) hold, then the series*


$$\begin{array}{c} \sum_{k=0}^{\infty }-\alpha_{k}g_{k}^{T}d_{k} \end{array}$$

*is finite.*

*Proof.* Since $\alpha_{k}>0$ is calculated by the strong Wolfe line search, then from (3) and (17) we obtain


$$\begin{array}{c} f(x_{k}+\alpha_{k}d_{k})-f(x_{k})\leq \psi \alpha_{k}g_{k}^{T}d_{k}\leq 0, \end{array}$$

or equivalently,


$$\begin{array}{c} f_{k+1}-f_{k}\leq \psi \alpha_{k}g_{k}^{T}d_{k}\leq 0. \end{array}$$ (21)

Therefore, the sequence $\left\{f_{k}\right\}$ is non-increasing, and the sequence $\left\{x_{k}\right\}$ remains within the level set Γ. According to Assumption 2.2, there exists a constant *f*^#^ such that $f_{k}$ converges to *f*^#^. Furthermore, according to the formal definition of sequence convergence, we obtain the following


$$\begin{array}{c} \lim_{k\to \infty }f_{k}=f^{\#}. \end{array}$$ (22)

We also have the following summation result:


$$\begin{array}{c} \sum_{k=0}^{\infty }(f_{k}-f_{k+1})=\lim_{n\to \infty }\sum_{k=0}^{n}(f_{k}-f_{k+1})=\lim_{n\to \infty }(f_{0}-f_{n+1}). \end{array}$$ (23)

By combining (22) and (23), it implies that


$$\begin{array}{c} \sum_{k=0}^{\infty }(f_{k}-f_{k+1})=f_{0}-f^{\#}<\infty , \end{array}$$ (24)

where $f_{0},f^{\#}$ are arbitrary constants. By multiplying (21) by −1, we obtain


$$\begin{array}{c} -\psi \alpha_{k}g_{k}^{T}d_{k}\leq f_{k}-f_{k+1}. \end{array}$$ (25)

From (24) and (25), it follows that


$$\begin{array}{c} \sum_{k=0}^{\infty }-\psi \alpha_{k}g_{k}^{T}d_{k}\leq \sum_{k=0}^{\infty }(f_{k}-f_{k+1})=f_{0}-f^{\#}<+\infty . \end{array}$$

Hence,


$$\begin{array}{c} \sum_{k=0}^{\infty }-\alpha_{k}g_{k}^{T}d_{k}<+\infty . \end{array}$$

□

**Lemma 2.4** *Suppose that Assumption 2.2 hold. Let* $\left\{x_{k}\right\}$ *be the sequence generated by the TTMZHS CG method with iteration form (2) where the search direction* $d_{k}$ *satisfies the descent condition (16) and the step size* $\alpha_{k}$ *is determined by the strong Wolfe line search conditions (3) and (4). Then, the Zoutendijk condition holds, that is,*


$$\begin{array}{c} \sum_{k=0}^{\infty }\frac{(g_{k}^{T}d_{k}{)}^{2}}{\|d_{k}{\|}^{2}}<\infty . \end{array}$$ (26)

*Proof.* From (4), we have


$$\begin{array}{c} \sigma g_{k}^{T}d_{k}\leq g_{k+1}^{T}d_{k}. \end{array}$$

Adding $-g_{k}^{T}d_{k}$ to both sides and applying the iteration formula (2), we obtain from (20) that


$$\begin{array}{cc} (\sigma -1)g_{k}^{T}d_{k} & \leq (g_{k+1}^{T}-g_{k}^{T})d_{k}=(g_{k+1}-g_{k}{)}^{T}d_{k}\leq |(g_{k+1}-g_{k}{)}^{T}d_{k}|\\ & \leq \|g_{k+1}-g_{k}\|\|d_{k}\|\leq L\|x_{k+1}-x_{k}\|\|d_{k}\|\\ & \leq L\|\alpha_{k}d_{k}\|=L\alpha_{k}\|d_{k}{\|}^{2}. \end{array}$$

Thus,


$$\begin{array}{c} \frac{(\sigma -1)g_{k}^{T}d_{k}}{L\|d_{k}{\|}^{2}}\leq \alpha_{k}. \end{array}$$

Multiplying both sides with $-g_{k}^{T}d_{k}>0$ (as guaranteed by Theorem 2.1), we obtain


$$\begin{array}{c} \frac{(1-\sigma )(g_{k}^{T}d_{k}{)}^{2}}{L\|d_{k}{\|}^{2}}\leq -\alpha_{k}g_{k}^{T}d_{k}, \end{array}$$

then,


$$\begin{array}{c} \frac{(g_{k}^{T}d_{k}{)}^{2}}{\|d_{k}{\|}^{2}}\leq -\frac{L}{\left(1-\sigma \right)}\alpha_{k}g_{k}^{T}d_{k}. \end{array}$$

Using Lemma 2.3 and the inequality above, we can conclude


$$\begin{array}{c} \sum_{k=0}^{\infty }\frac{(g_{k}^{T}d_{k}{)}^{2}}{\|d_{k}{\|}^{2}}\leq \frac{L}{1-\sigma }\sum_{k=0}^{\infty }(-\alpha_{k}g_{k}^{T}d_{k})<+\infty . \end{array}$$ (27)

Hence, the Zoutendijk condition (26) holds.□

#### 2.2.2 Global convergence properties.

Global convergence is an important property in the analysis of optimization algorithms. It means the method generates an iterative sequence that converges to a stationary point, regardless of the initial starting point. Specifically, from any *x*_0_, the algorithm produces a sequence $\left\{x_{k}\right\}$ with a subsequence converging to $x^{*}$ such that $g(x^{*})=0$ [26].

Mathematically, a commonly used criterion for global convergence is the following.


$$\begin{array}{c} \underset{k\to \infty }{lim inf}\|g_{k}\|=0. \end{array}$$ (28)

This condition ensures a subsequence exists whose gradient norm tends to zero, showing the algorithm does not diverge from stationary points [26].

Building on the global convergence framework outlined above, the following theorem establishes the convergence properties of the TTMZHS CG method with a strong Wolfe line search.

**Theorem 2.5.** *Let the sequence* $\left\{x_{k}\right\}$ *be generated by Algorithm 1. Assume the objective function f is continuously differentiable and bounded below,* $d_{k}$ *is a descent direction, and the step size* $\alpha_{k}$ *satisfies the strong Wolfe conditions (3) and (4). Then, the global convergence condition (28) holds.*

*Proof.* This theorem uses contradiction. If (28) fails, then there exists an *h* > 0 such that


$$\begin{array}{c} \|g_{k}\|>h,\;\forall k\geq 0. \end{array}$$ (29)

From the form of $\beta_{k}^{MZHS}$, for all k≥1, the value satisfies


$$\begin{array}{cc} \left|\beta_{k}^{MZHS}\right| & =\left|\frac{g_{k}^{T}y_{k-1}}{\max\left\{\eta \|d_{k-1}\|\|y_{k-1}\|,-g_{k}^{T}d_{k-1},\|g_{k-1}{\|}^{2}\right\}}\right|\\ & \leq \frac{\left\|g_{k}\|\|y_{k-1}\right\|}{\eta \|d_{k-1}\|\|y_{k-1}\|}\\ & \leq \frac{\left\|g_{k}\right\|}{\eta \|d_{k-1}\|}. \end{array}$$ (30)

From (14), we get


$$\begin{array}{cc} \left\|d_{k}\right\| & =\|-g_{k}+\beta_{k}^{\text{MZHS}}d_{k-1}+\theta_{k}^{\text{MZHS}}y_{k-1}\|\\ & \leq \|g_{k}\|+|\beta_{k}^{\text{MZHS}}|\|d_{k-1}\|+|\theta_{k}^{\text{MZHS}}|\|y_{k-1}\|\\ & \leq \|g_{k}\|+\frac{\left\|g_{k}\right\|}{\eta \|d_{k-1}\|}\|d_{k-1}\|+\left|\frac{g_{k}^{T}d_{k-1}}{\max\left\{\eta \|d_{k-1}\|\|y_{k-1}\|,-g_{k}^{T}d_{k-1},\|g_{k-1}{\|}^{2}\right\}}\right|\|y_{k-1}\|\\ & \leq \left(1+\frac{1}{\eta }\right)\|g_{k}\|+\frac{\left|g_{k}^{T}d_{k-1}\right|}{\eta \|d_{k-1}\|\|y_{k-1}\|}\|y_{k-1}\|\\ & \leq \left(1+\frac{1}{\eta }\right)\|g_{k}\|+\frac{\left\|g_{k}\|\|d_{k-1}\right\|}{\eta \|d_{k-1}\|\|y_{k-1}\|}\|y_{k-1}\|=\left(1+\frac{2}{\eta }\right)\|g_{k}\|\\ & \leq \left(1+\frac{2}{\eta }\right)\zeta =T, \end{array}$$

where the second inequality is based on properties of the triangle inequality and the absolute homogeneity of the norm, the third inequality from (30) and (14), the fourth inequality from the property of absolute value, the fifth inequality from Cauchy-Schwarz Inequality, and the final inequality based on (19). From above, it can be concluded that the norm of the search direction of the TTMZHS CG method has an upper bound, that is,


$$\begin{array}{c} \|d_{k}\|\leq T,\;\forall k\geq 0, \end{array}$$ (31)

where *T* is an arbitrary constant. Furthermore, (29) and (31), we obtain


$$\begin{array}{c} \frac{\left\|g_{k}\right\|}{\left\|d_{k}\right\|}>\frac{h}{T}. \end{array}$$

That implies


$$\begin{array}{c} \frac{\|g_{k}{\|}^{4}}{\|d_{k}{\|}^{2}}>\frac{h^{4}}{T^{2}}. \end{array}$$

Since $g_{k}^{T}d_{k}=-\|g_{k}{\|}^{2}$, it follows


$$\begin{array}{c} \frac{(g_{k}^{T}d_{k}{)}^{2}}{\|d_{k}{\|}^{2}}>\frac{h^{4}}{T^{2}}. \end{array}$$

Taking summation over *k* = 0 to *n*:


$$\begin{array}{c} \sum_{k=0}^{n}\frac{(g_{k}^{T}d_{k}{)}^{2}}{\|d_{k}{\|}^{2}}>\sum_{k=0}^{n}\frac{h^{4}}{T^{2}}=\frac{h^{4}}{T^{2}}(n+1). \end{array}$$

Letting n→∞, we obtain


$$\begin{array}{c} \sum_{k=0}^{\infty }\frac{(g_{k}^{T}d_{k}{)}^{2}}{\|d_{k}{\|}^{2}}>\lim_{n\to \infty }\frac{h^{4}}{T^{2}}(n+1)=\infty . \end{array}$$

This result contradicts Zoutendijk’s condition (26) and thus completes the proof. Therefore, global convergence, as stated in (28), must hold for the TTMZHS CG method.

## 3 Numerical reports

This section discusses the numerical performance of the TTMZHS CG method using various test functions. A total of 273 problems with varying dimensions were used. The list of test functions used is based on those listed by More et al. [30], Andrei [31], Jamil & Yang [32], and Andrei [26], and is shown in Table 1.

**Table 1:** List of test functions.

No.	Function/Dimension	No.	Function/Dimension	No.	Function/Dimension
1	COSINE/6000	92	BV/2000	183	BOOTH/2
2	COSINE/100000	93	BV/20000	184	BOOTH/2
3	COSINE/800000	94	IE/500	185	TRECANNI/2
4	DIXMAANA/6000	95	IE/1500	186	TRECANNI/2
5	DIXMAANA/90000	96	SING/1000	187	ZETTL/2
6	DIXMAANB/24000	97	SING/2000	188	ZETTL/2
7	DIXMAANB/48000	98	LINEAR/100	189	SHALLOW/1000
8	DIXMAANC/2700	99	LINEAR/500	190	SHALLOW/50000
9	DIXMAANC/27000	100	OSBORNE2/11	191	SHALLOW/100000
10	DIXMAAND/12000	101	PENALTY1/200	192	GENQUARTIC/100
11	DIXMAAND/90000	102	PENALTY1/1000	193	GENQUARTIC/5000
12	DIXMAANE/2400	103	PENALTY2/100	194	GENQUARTIC/10000
13	DIXMAANE/48000	104	PENALTY2/110	195	QDRTCQF2/10
14	DIXMAANF/15000	105	EXROSE/500	196	QDRTCQF2/100
15	DIXMAANF/60000	106	EXROSE/1000	197	QDRTCQF2/1000
16	DIXMAANG/12000	107	TRID/500	198	LEON/2
17	DIXMAANG/90000	108	TRID/50	199	LEON/2
18	DIXMAANH/6000	109	HIMMELH/70000	200	GENTRID1/5
19	DIXMAANH/150000	110	HIMMELH/240000	201	GENTRID1/10
20	DIXMAANI/360	111	BADSCB/2	202	GENTRID1/100
21	DIXMAANI/36	112	BD/4	203	GENTRID2/10
22	DIXMAANJ/3000	113	BIGGS/6	204	GENTRID2/50
23	DIXMAANJ/15000	114	OSBORNE1/5	205	GENTRID2/500
24	DIXMAANK/12000	115	EXBEALE/5000	206	POWER1/10
25	DIXMAANK/120	116	EXBEALE/10000	207	POWER1/50
26	DIXMAANL/2400	117	HIMMELBC/500000	208	POWER1/500
27	DIXMAANL/24000	118	HIMMELBC/1000000	209	QDRTCQF1/100
28	DIXON3DQ/150	119	ARWHEAD/100	210	QDRTCQF1/1000
29	DIXON3DQ/15	120	ARWHEAD/1000	211	QDRTCQF1/10000
30	DQDRTIC/9000	121	ENGVAL1/500000	212	QP2/5
31	DQDRTIC/90000	122	ENGVAL1/1000000	213	QP2/50
32	QUARTICM/5000	123	DENSCHNA/500000	214	QP2/500
33	QUARTICM/150000	124	DENSCHNA/1000000	215	QP1/5
34	EDENSCH/7000	125	DENSCHNB/500000	216	QP1/10
35	EDENSCH/40000	126	DENSCHNB/1000000	217	QP1/100
36	EDENSCH/500000	127	DENSCHNC/10	218	QUARTIC/4
37	EG2/100	128	DENSCHNC/500	219	QUARTIC/4
38	EG2/35	129	DENSCHNF/500000	220	MATYAS/2
39	FLETCHCR/1000	130	DENSCHNF/1000000	221	MATYAS/2
40	FLETCHCR/50000	131	ENGVAL8/500000	222	COLVILLE/4
41	FLETCHCR/200000	132	ENGVAL8/1000000	223	COLVILLE/4
42	FREUROTH/460	133	WHITE/10	224	DIXPRICE/1000
43	FREUROTH/10	134	BDQRTIC/10	225	DIXPRICE/10000
44	GENROSE/10000	135	DIAGONAL6/100	226	DIXPRICE/100000
45	GENROSE/100	136	EXWHOL/50000	227	SPHERE/1000
46	HIMMELBG/70000	137	EXWHOL/100000	228	SPHERE/10000
47	HIMMELBG/240000	138	EXWHOL/1000000	229	SPHERE/100000
48	LIARWHD/15	139	EXROSE/50000	230	SUMSQR/1000
49	LIARWHD/1000	140	EXROSE/100000	231	SUMSQR/10000
50	PENALTY1/1000	141	EXROSE/1000000	232	SUMSQR/50000
51	PENALTY1/8000	142	EXFRERTH/1000	233	DENSCHNA/1000
52	QUARTC/4000	143	EXFRERTH/50000	234	DENSCHNA/10000
53	QUARTC/80000	144	EXFRERTH/100000	235	DENSCHNA/100000
54	QUARTC/500000	145	EXBEALE/1000	236	DENSCHNC/100
55	TRIDIA/300	146	EXBEALE/50000	237	DENSCHNC/5000
56	TRIDIA/50	147	EXBEALE/100000	238	DENSCHNC/50000
57	EXWOOD/150000	148	RAYDAN1/10	239	BD1/100
58	EXWOOD/200000	149	RAYDAN1/50	240	BD1/5000
59	BDEXP/5000	150	RAYDAN1/100	241	BD1/50000
60	BDEXP/50000	151	EXTRID1/10	242	HIMMELH/10
61	BDEXP/500000	152	EXTRID1/50	243	HIMMELH/50
62	DENSCHNF/90000	153	EXTRID1/100	244	HIMMELH/100
63	DENSCHNF/280000	154	DIAGONAL4/1000	245	HIEBERT/1000
64	DENSCHNF/600000	155	DIAGONAL4/5000	246	HIEBERT/10000
65	DENSCHNB/6000	156	DIAGONAL4/50000	247	HIEBERT/100000
66	DENSCHNB/24000	157	EXHIMBL/1000	248	ENGVAL1/5
67	DENSCHNB/300000	158	EXHIMBL/50000	249	ENGVAL1/10
68	GENQUARTIC/9000	159	EXHIMBL/100000	250	ENGVAL1/50
69	GENQUARTIC/90000	160	FLETCHCR/100	251	ENGVAL8/5
70	GENQUARTIC/500000	161	FLETCHCR/5000	252	ENGVAL8/10
71	BIGGSB1/110	162	EXPOW/100	253	ENGVAL8/50
72	BIGGSB1/200	163	EXPOW/1000	254	LINPERT/100
73	SINE/100000	164	NONSCOMP/2	255	LINPERT/5000
74	SINE/50000	165	NONSCOMP/4	256	LINPERT/50000
75	FLETCBV/15	166	NONSCOMP/10	257	QUARTICM/1000
76	FLETCBV/55	167	DENSCHNB/1000	258	QUARTICM/50000
77	NONSCOMP/5000	168	DENSCHNB/50000	259	QUARTICM/100000
78	NONSCOMP/80000	169	DENSCHNB/100000	260	DDBAU/10
79	POWER1/150	170	EXPENU52/5	261	DDBAU/500
80	POWER1/90	171	EXPENU52/10	262	DDBAU/5000
81	RAYDAN1/500	172	EXPEN/50	263	HARKERP/1000
82	RAYDAN1/5000	173	HAGER/5	264	HARKERP/50000
83	RAYDAN2/2000	174	HAGER/10	265	HARKERP/100000
84	RAYDAN2/20000	175	HAGER/50	266	QP3/5
85	RAYDAN2/500000	176	EXMAR/10	267	QP3/10
86	DIAGONAL1/800	177	EXMAR/50	268	QP3/100
87	DIAGONAL1/2000	178	EXMAR/100	269	DIAGAUP1/10
88	DIAGONAL2/100	179	SIXHUMP/2	270	DIAGAUP1/1000
89	DIAGONAL2/1000	180	SIXHUMP/2	271	DIAGAUP1/10000
90	DIAGONAL3/500	181	THREEHUMP/2	272	ARWHEAD/10
91	DIAGONAL3/2000	182	THREEHUMP/2	273	ARWHEAD/50

Numerical experiments were conducted to compare the performance of the TTMZHS CG method with its predecessor methods, namely, the ZHS and TTRMIL conjugate gradient methods. In addition, the proposed method is compared with two well-established nonlinear conjugate gradient solvers, namely the ALG [22] and TTRMIL+ [33] methods. All parameters used in the compared methods were maintained according to their original specifications as reported in the corresponding literature.

The numerical experiment tracked the number of iterations (NOI), computation time (CPU), number of gradient calculations (NOG), and number of test function calculations (NOF). NOI measures the algorithm’s convergence rate. CPU time reflects computational efficiency. NOG counts required gradient computations, and NOF records objective function evaluations, usually during line search or convergence checks. These criteria offer a comprehensive view of the algorithm’s performance and efficiency.

The numerical experiments were conducted in MATLAB on a personal laptop running Microsoft Windows 10, an Intel Core i7 processor, and 32GB of RAM. We evaluated three methods on 273 test functions in various dimensions (2≤n≤1,000,000). The stopping criterion was $\epsilon <10^{-6}$. Strong Wolfe parameters were ψ=0.0001 and σ=0.001, with a maximum of 2000 iterations. The value of ψ ensures a sufficient decrease in the objective function. The smaller σ imposes a stricter curvature condition, enhancing stability and supporting reliable convergence of the proposed method. A failure (F) is recorded when the line search terminates before satisfying the convergence tolerance for $\|g(x_{k})\|<\epsilon$.

The numerical results of the TTMZHS CG method and its base methods, ZHS, TTRMIL, ALG, and TTRMIL + , for solving 273 problems are presented in a file accessible at: https://bit.ly/4oViKjR. The MATLAB implementation code of the proposed method is available upon request.

To provide a clear summary, Table 2 presents the number of test functions successfully completed by each method based on the numerical results.

**Table 2:** Summary of numerical experiment results.

Method	Success	Fail
ZHS	219	54
TTRMIL	197	76
ALG	165	108
TTRMIL+	190	83
TTMZHS	230	43

Table 2 summarizes the overall performance of the tested algorithms. Among the five methods, the TTMZHS CG method achieved the best performance by successfully solving 230 out of 273 test problems, corresponding to 87.91%. The ZHS CG method solved 219 problems (80.22%), followed by the TTRMIL CG method with 197 solved problems (72.16%). Meanwhile, the TTRMIL+ and ALG CG methods solved 190 (69.60%) and 165 (60.44%) problems, respectively. These results indicate that TTMZHS is more robust and reliable compared with the other competing methods.

To gain a clearer understanding of the performance of the TTMZHS CG method compared with the ZHS, TTRMIL, ALG, and TTRMIL+ CG methods, a visual comparison based on performance profiles is employed.

The results of the numerical experiments can be visualized using a performance profile curve, as introduced by Dolan and Moré [34]. This concept provides a robust framework for assessing and comparing the performance of a set of solvers *S* across a set of test problems *P*.

Suppose there are $n_{s}$ solvers and $n_{p}$ test problems. For each problem p∈P and solver s∈S, let $t_{p,s}$ denote the computational effort required by solver *s* to solve problem *p*—this can be measured by CPU time, NOF, NOG, or NOI.

To normalize performance across problems, a performance ratio is computed as:


$$\begin{array}{c} r_{p,s}=\frac{t_{p,s}}{\min\{t_{p,s}:s\in S\}}. \end{array}$$

This ratio compares the performance of solver *s* on problem *p* with the best-performing solver on that problem. The performance profile for solver *s* is then defined as:


$$\begin{array}{c} \rho_{s}(\tau )=\frac{1}{n_{p}}\cdot \text{ size}\left\{p\in P:r_{p,s}\leq \tau \right\}, \end{array}$$

where τ≥1 is a user-defined performance threshold. The function $\rho_{s}(\tau )$ gives the fraction of problems for which solver *s* performs within a factor τ of the best solver. A higher value of $\rho_{s}(\tau )$ indicates better overall performance. A solver with $\rho_{s}(\tau )\approx 1$ for small τ values is considered more efficient and reliable.

**Fig 1:**
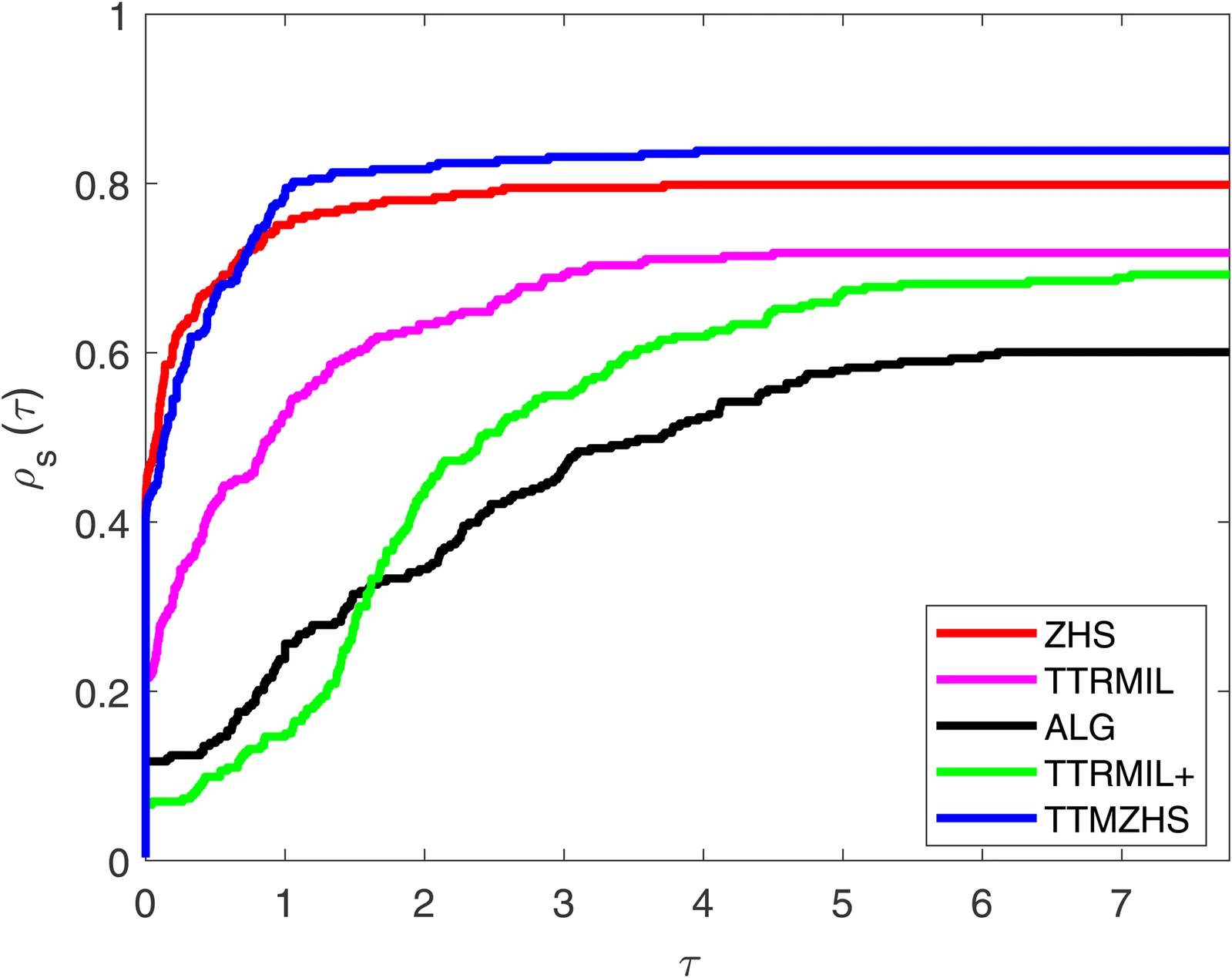
Performance metrics for NOI. The axes represent the NOI performance ratio and the proportion of problems solved.

**Fig 2:**
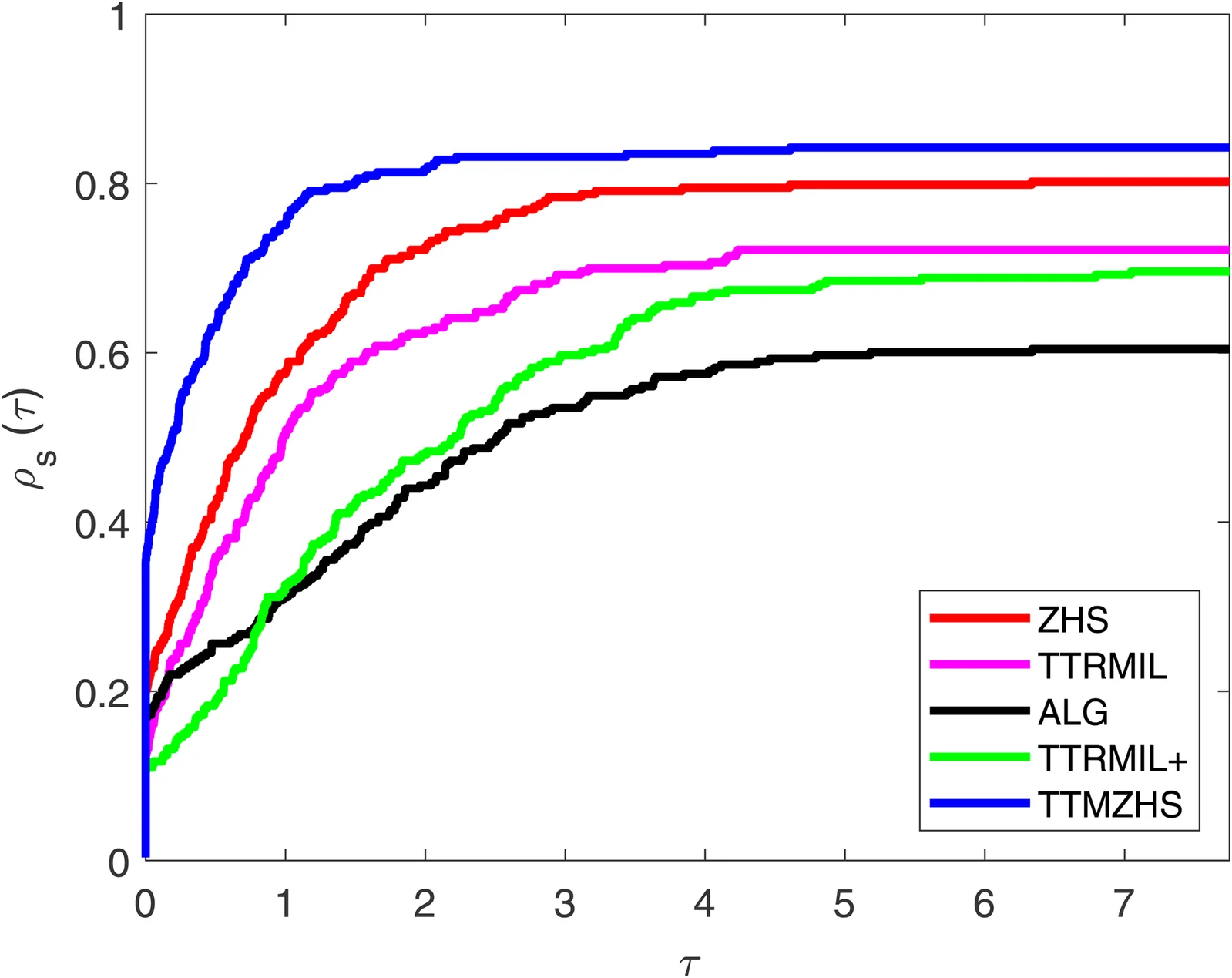
Performance metrics for CPU Time. The axes represent the CPU time performance ratio and the proportion of problems solved.

**Fig 3:**
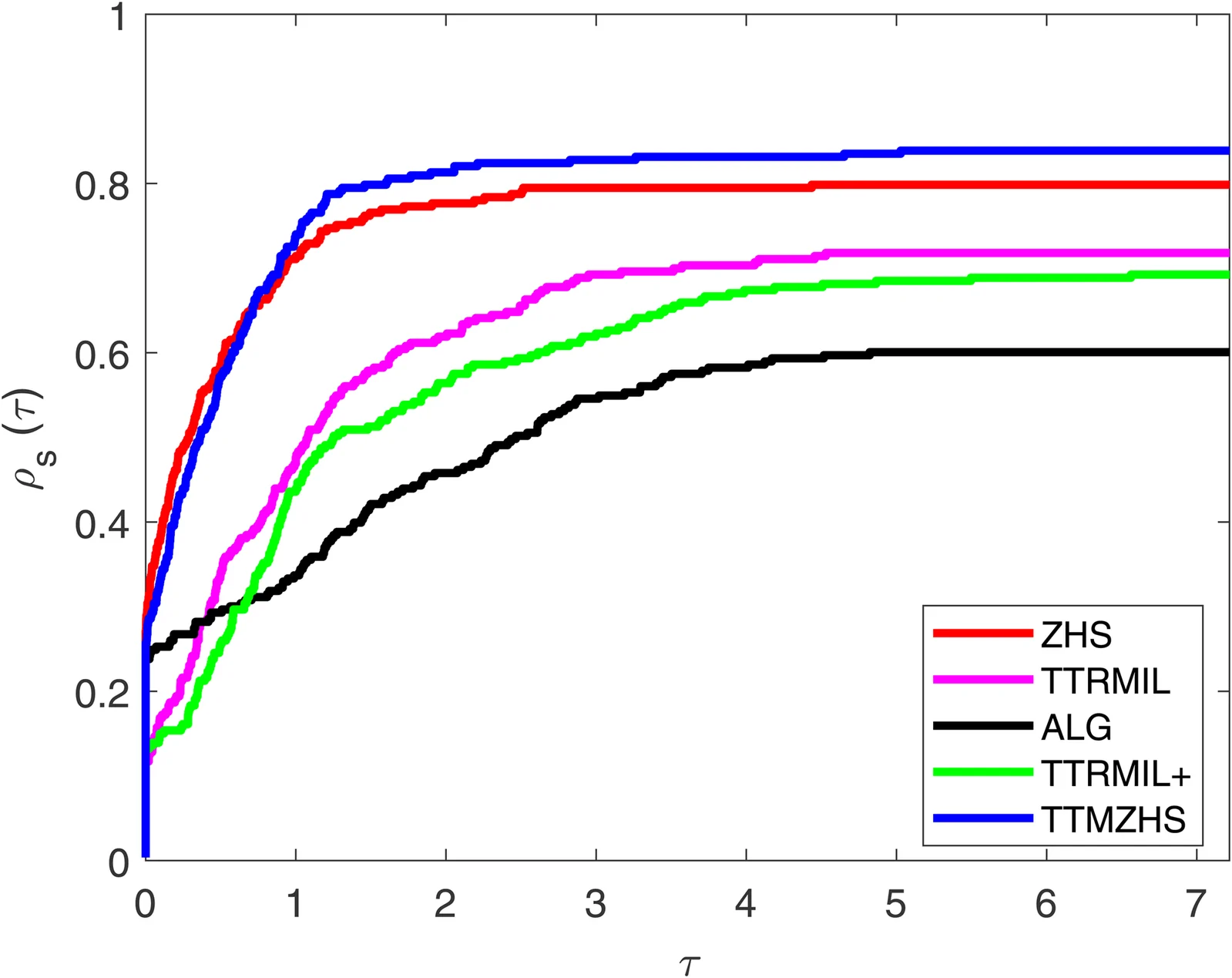
Performance metrics for NOF. The axes represent the NOF performance ratio and the proportion of problems solved.

**Fig 4:**
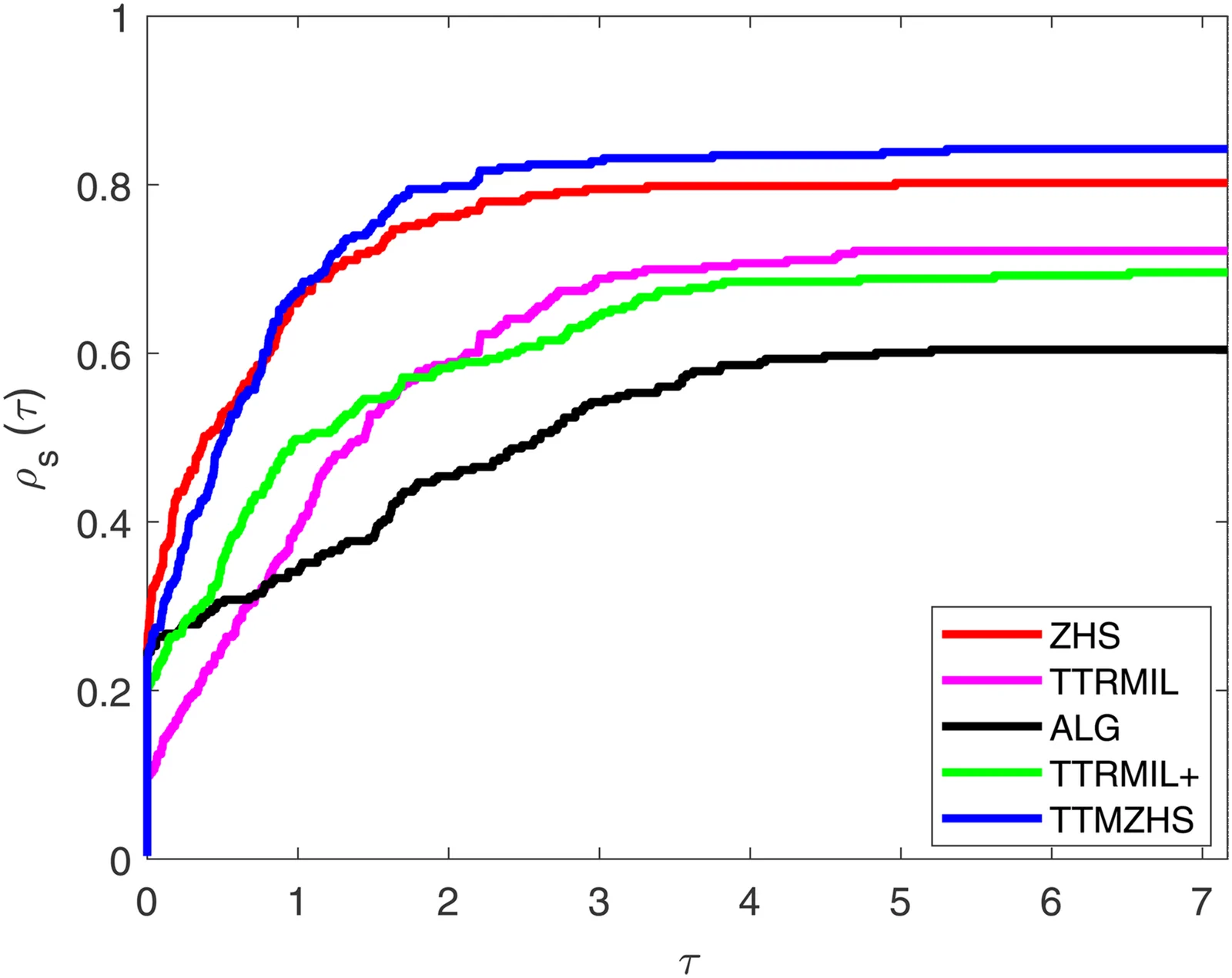
Performance metrics for NOG. The axes represent the NOG performance ratio and the proportion of problems solved.

Based on the numerical results, the performance profiles of the ZHS, TTRMIL, ALG, TTRMIL + , and TTMZHS CG methods are presented in Figs 1–4 with respect to four performance measures: NOI, CPU time, NOF, and NOG.

Fig 1 presents the performance profile with respect to NOI. Across most values of the performance ratio τ, the proposed TTMZHS CG method consistently outperforms the others. As a result, it solves a larger proportion of the test problems with fewer iterations.

This consistent outperformance continues for CPU time, as illustrated in Fig 2. While the performance curve of TTMZHS rises more rapidly and remains above those of the other methods for most values of τ, it also indicates that the proposed method solves a greater percentage of problems within shorter computational time.

A similar pattern emerges in the number of function evaluations (Fig 3): TTMZHS CG method again performs better, solving more problems at lower evaluation thresholds. This result reflects its improved computational efficiency.

Finally, Fig 4 presents the performance profile for gradient evaluations: the TTMZHS CG method not only continues to outperform the other algorithms, but also solves a larger proportion of problems and requires fewer gradient evaluations.

In general, TTMZHS CG method consistently demonstrates robustness and computational efficiency across all four performance measures for large-scale unconstrained optimization problems.

## 4 Applications in the low carbon supply chain optimization

Optimization problems in the LCSC optimization problem are challenging because the economic, environmental, and operational decision variables interact nonlinearly. Pricing decisions, warranty policies, and carbon-emission reduction levels all affect market demand, production costs, and emission-trading outcomes. These interactions create objective functions that are nonlinear and continuously differentiable, with multiple coupled decision variables. Large-scale LCSC optimization problem usually lack closed-form solutions and require iterative numerical methods. CG methods work well in this context. They offer strong convergence, low memory use, and solve nonlinear unconstrained problems without needing second-order derivatives. These features make CG-type methods well-suited to practical LCSC optimization problem [35].

In this part, we apply the TTMZHS CG method specifically to the Low Carbon Supply Chain Optimization Problem. Here, the method determines the optimal set of decision variables—pricing, warranty, and carbon-reduction levels—that maximizes profits for all supply chain members in the context of LCSC optimization problem. The solution process directly addresses the interaction of these variables within the LCSC framework, employing the approach described by Zhou et al. [35].

LCSC optimization problem is a major topic in supply chain research, motivated by stricter regulations and a growing interest in eco-friendly products. Companies must reduce carbon emissions while remaining competitive in both costs and service quality. Building on this context, our study applies the TTMZHS algorithm to real-world supply chain scenarios to enhance three key areas: pricing, warranty policy, and carbon emission reduction. By evaluating these elements together, our model demonstrates the balance among efficiency, customer satisfaction, and sustainability. As a result, companies can act responsibly for the environment while staying profitable, providing a clear guide for sustainable supply chain management [36].

**Fig 5:**
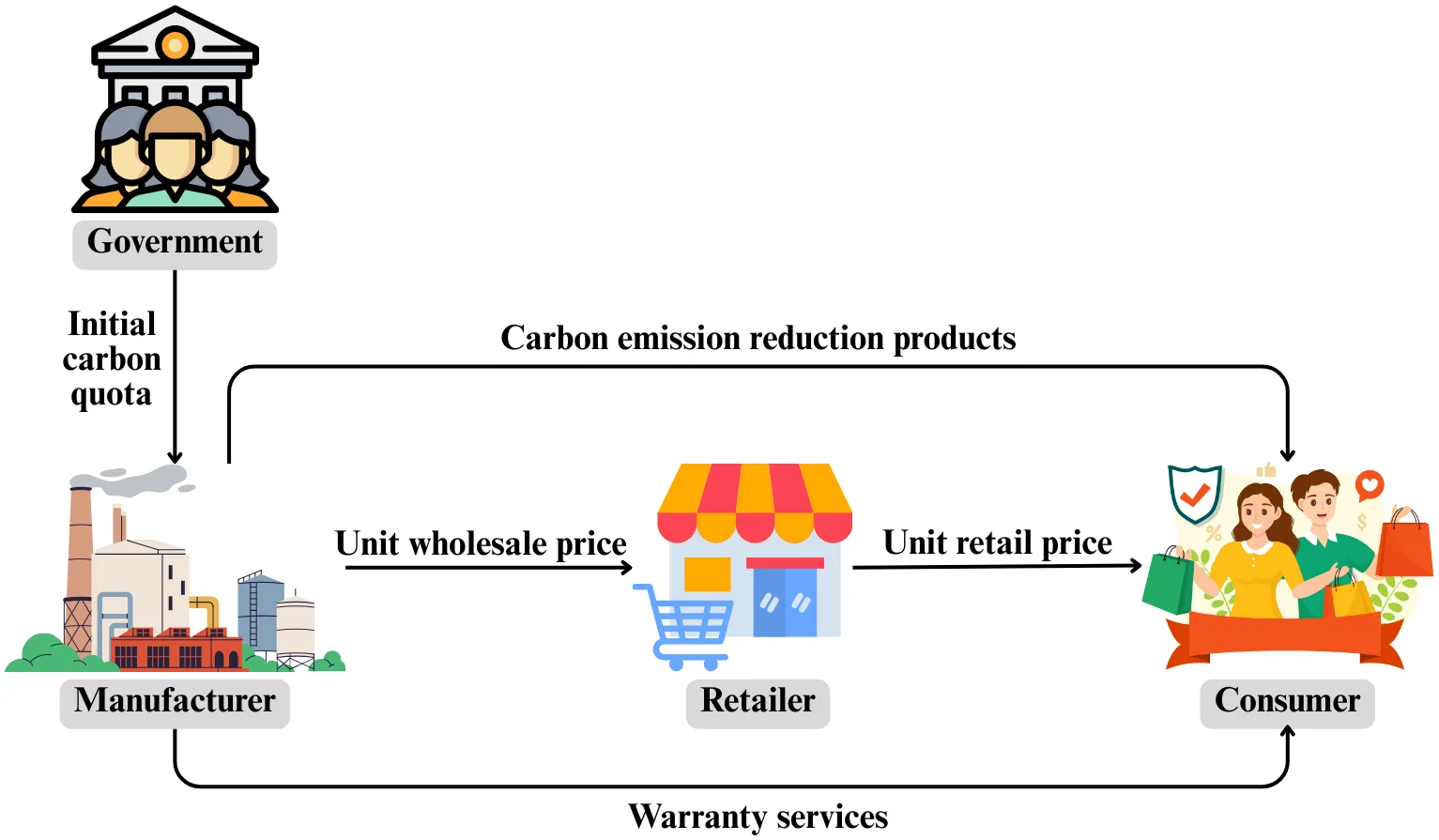
Low-carbon supply chain operation flow chart. The chart shows the interactions among the supply chain participants.

As shown in Fig 5, manufacturers distribute products through retailers and provide warranty services to consumers for product repairs. Both the production process and the provision of warranty services contribute to carbon emissions, which in turn impact the environment. To mitigate this effect, the government imposes carbon-emission quotas that all companies must comply with. Companies whose emissions exceed their assigned quota must purchase additional allowances, while those emitting below their quota may sell their surplus in the carbon emissions trading market, thereby balancing overall emissions.

The LCSC optimization problem is often modeled using a multi-echelon framework. This helps capture how different stakeholders interact. Qu et al. [37] studied a two-echelon supply chain with manufacturers and retailers. Based on the Stackelberg theory, they built a game model. In the model, retail prices, warranty periods, and carbon emission reductions affect consumer demand. Qu et al. [37] also proposed a centralized policy model. This model serves as the basis for our optimization framework. Here, the manufacturer’s profit is $\Pi_{M}^{Co}$, the retailer’s profit is $\Pi_{R}^{Co}$, and their total or joint profit is $\Pi_{T}^{Co}$.

The profit function model for manufacturers combines several factors that affect profits. These include revenue from new product sales, carbon emissions trading, and costs related to production, emission reduction, and warranty investments. To clarify how these elements interact, it is important to note that manufacturers must balance sales and carbon emissions revenue with the costs of emission reduction and warranty provision. The profit function is


$$\begin{array}{c} \Pi_{M}^{Co}(\omega ,\lambda ,\theta )=(\omega -c_{m})D+k(a-eD-\varepsilon eR)-\frac{1}{2}u\lambda^{2}-b\theta^{2}. \end{array}$$ (32)

To clarify the role of retailers in the overall profit analysis, the retailer profit function shows that profits depend on the difference between the selling price and the purchase price per unit, as well as on sales volume, which is influenced by market demand. The profit function for retailers is


$$\begin{array}{c} \Pi_{R}^{Co}(p)=(p-\omega )D. \end{array}$$ (33)

To transition from the individual profit models to the overall optimization, we combine the $\Pi_{M}^{Co}$ and $\Pi_{R}^{Co}$ models to obtain the $\Pi_{T}^{Co}$ total profit function for LCSC. The resulting LCSC optimization problem is given by:


$$\begin{array}{c} \max_{p,\lambda ,\theta }\;\Pi_{T}=(p-c_{m})D+k(a-eD-\varepsilon eR)-\frac{1}{2}u\lambda^{2}-b\theta^{2}. \end{array}$$ (34)

To enhance clarity, Table 3 summarizes all symbols used in the model.

**Table 3:** Symbolic explanation table.

$c_{m}$	: Unit production cost of new product
*k*	: Unit carbon emissions trading price
*a*	: Initial amount of carbon emissions allocated by the government
*e*	: Carbon emissions from the production of one unit of new product
*u*	: Carbon emission reduction investment cost coefficient
*b*	: Warranty period investment cost coefficient
*D*	: Market demand
*R*	: Total number of products returned for repair
$\Pi_{s}^{i}$	: Profit function of member *s* in mode *i*
*p*	: Unit retail price
θ	: Warranty period
ω	: Unit wholesale price
λ	: Carbon emission reduction level of product
ε	: Ratio of carbon emissions

The TTMZHS algorithm, together with ZHS, TTRMIL, ALG, and TTRMIL + , is applied to the LCSC optimization problem to solve (34). Random initial values are assigned to the unit retail price, warranty period, and carbon-emission-reduction level of the product to provide a fair and comprehensive comparison of the algorithms in terms of convergence performance and computational efficiency.

Next, the parameter value is set at *k* = 9.1, corresponding to the average price of carbon emission trading units in Fujian Province, China, during April 2020. With reference to relevant literature [38–40], other parameters are set as follows: $c_{m}=500$, *a* = 5000, *e* = 10, ε=0.3, *u* = 15, *b* = 10, D=300−0.12p+0.84θ+0.8λ, and R=50+0.12θ. With these parameters in place, the optimal values obtained for the LCSC optimization problem solved using the conjugate gradient methods of ZHS, TTRMIL, ALG, TTRMIL + , AND TTMZHS are (p,θ,λ)=(2563.6,82.7,105.2) and the respective values of the NOI, CPU time, NOF, and NOG metrics are stated in Table 4.

**Table 4:** Comparison of methods for the LCSC optimization problem.

Method	CPU Time	NOF	NOG	NOI
ZHS	0.033788	361	267	90
TTRMIL	0.118290	1346	1213	142
ALG	0.0999	996	723	202
TTRMIL+	0.4139	4298	3021	1812
TTMZHS	0.026342	277	177	28

Based on Table 4, there are clear differences in performance among the five methods. The TTMZHS CG method requires the least CPU time, the smallest functions and gradient evaluations, and the fewest iterations compared to ZHS, TTRMIL, ALG, and TTRMIL + . These results indicate that TTMZHS is the most efficient method for solving the optimization problem of LCSC.

In terms of its economic and operational implications, the optimal solution (p,θ,λ)=(2563.6,82.7,105.2) can be interpreted within the context of the LCSC decision-making problem. Specifically, *p* = 2563.6 represents the optimal unit retail price that balances the revenue obtained from product sales against the reduction in market demand caused by price sensitivity. The optimal warranty period, θ=82.7, indicates the warranty level that provides a sufficient incentive for the customer to increase market demand while accounting for the additional costs associated with the warranty provision and the repair of the product. Meanwhile, λ=105.2 represents the optimal level of carbon emission-reduction, reflecting the trade-off between the benefits of reducing emissions, including its positive effect on market demand and carbon emissions trading outcomes, and the associated investment cost of emission reduction. Therefore, the solution obtained represents the combination of pricing, warranty and carbon-emission reduction decisions that optimally balance the economic and environmental considerations incorporated in the LCSC model.

## 5 Conclusion

In this paper, we introduce TTMZHS, a three-term conjugate gradient method for unconstrained optimization that integrates principles from the ZHS and TTRMIL parameterizations. We establish its validity by showing it meets sufficient descent and global convergence under strong Wolfe line search. Numerical experiments verify its superior efficiency versus existing CG methods with similar features. We demonstrate its value in a real-world scenario—optimizing carbon emission reductions in a low-carbon supply chain model. TTMZHS surpasses ZHS, TTRMIL, ALG, and TTRMIL+ in optimizing the LCSC optimization problem, using less CPU time, fewer evaluations, and converging in fewer iterations. These results confirm its robust theoretical foundation and practical efficacy for advancing carbon reduction in supply chain systems.
