# Supplementary material for: A three-term conjugate gradient method with a new descent structure for unconstrained optimization and low-carbon supply chain optimization
Source: PLoS One. 2026 Aug 27;21(8):e0356498. doi: 10.1371/journal.pone.0356498 (PMC13521400; doi:10.1371/journal.pone.0356498)
Supplement: Supplementary file 1 [file pone.0356498.s002.pdf]

Manuscript ID: *PONE-D-25-55566*

Title: *A Three-Term Conjugate Gradient Method with a New Descent Structure for Unconstrained Optimization and Low-Carbon Supply Chain Optimization*

Response to Reviewer 1, Reviewer 2, and Reviewer 3

We thank the reviewer for his/her careful reading, and for raising relevant concerns. We tried our best to correct all the misprints we observe during the revision. Below we replicate the reviewer's comments and provide our specific responses. For easy checking of our changes in the text, the modifications are highlighted in red.

Reviewer 1: The manuscript presents a new hybrid three-term conjugate gradient method (TTMZHS) for solving unconstrained optimization problems. The authors establish sufficient descent and global convergence properties under the strong Wolfe line search and validate the method through comprehensive numerical experiments on 273 benchmark functions. The inclusion of a Low Carbon Supply Chain (LCSC) optimization example demonstrates the practical relevance and applicability of the proposed approach.

The manuscript offers a meaningful contribution to the field of numerical optimization and is supported by strong theoretical and computational results. I believe the paper is fundamentally sound and suitable for publication in PLOS ONE after minor revisions primarily concerning writing clarity and small improvements in exposition.

Minor comments:

Comment 1: Language and Grammar: Please carefully proofread the manuscript to correct grammatical errors and improve readability. For example, the Abstract sentence "In this paper proposes..." should be revised to "This paper proposes...". Several similar issues appear throughout the text.

Response: Thank you for the suggestion. The manuscript has been carefully proofread to correct grammatical errors, improve readability, and ensure clarity throughout the text (using grammarly). The sentence in the Abstract and other similar issues have been revised accordingly..

Comment 2: Motivation for the Hybrid Method: In Section 2.1, briefly clarify why TTZHS and TTRMIL were chosen to be combined. A short explanation of the design intuition behind TTMZHS would help readers follow the logic.

Response: Thank you for the valuable comment. We have revised Section 2.1 to clarify the motivation behind the proposed hybrid method. In particular, we explained that TTZHS provides strong stability and descent properties through its safeguarded denominator, while TTRMIL offers flexibility and improved search direction through its three-term structure. The proposed TTMZHS method combines these advantages to improve robustness and convergence performance..

Comment 3: Low Carbon Supply Chain Application: The transition to the LCSC model is abrupt. Please add a brief introductory paragraph explaining why this problem is challenging and why CG-type methods are appropriate.

Response: Thank you for the helpful suggestion. We have added an introductory paragraph at the beginning of Section 4 explaining the challenges of the LCSC optimization problem and why CG-type methods are suitable, due to their effectiveness in solving nonlinear unconstrained optimization problems efficiently.

Reviewer 2: The manuscript proposed a modified three-term conjugate gradient (CG) method for unconstrained smooth optimization, named TTMZHS. The new method focuses on the denominators of two scalars. Comprehensive analysis was conducted to support the theoretical properties of convergence, and showed empirical superior over two other three term methods. A case study applying the method to a low-carbon supply chain profit optimization model that shows superior performance.

Strength: 1. Clear model definition. 2. Comprehensive convergence analysis. 3. Comprehensive analysis over a large benchmark set. 4. Application is aligned with the scope of Plos One.

Major comments:

Comment 1: Novelty is limited, as the paper combines components from prior TTZHS and TTRMIL directions. Beyond performance, theoretical justification would be help to distinguish from existing methods.

Response: Thank you for this important comment. In response, we have strengthened the theoretical justification of the proposed TTMZHS method. In particular, we clarified the theoretical motivation of the hybrid parameter design and emphasized the role of the safeguarded denominator in improving numerical stability and preserving descent properties. These modifications help distinguish the proposed method from existing TTZHS and TTRMIL methods both theoretically and structurally.

Comment 2: Insufficient literature review with limited benchmarks. Only two close relatives (TTZHS and TTRMIL) are compared. CG is widely studied and used in the literature. For example, there is a follow up paper of TTRMIL published in 2020 "Some three-term conjugate gradient methods with the new direction structure." Well-established nonlinear CG like Hager–Zhang is also omitted in the literature review and experiment, while its test function is included in the experiment. A more comprehensive review is needed as well.

Response: We have cited the HZ method and we have also added numerical experiments of the RMIL+ three-term method as well as the latest three-term method in 2025.

Comment 3: The numerical section states strong Wolfe constants psi=$10^{-}4$ and sigma=0.001. However, in typical strong Wolfe implementations, sigma is much larger (also see the 2020 paper mentioned above). Justification of this choice, and an additional sensitivity analysis is needed to justify the choice of parameters.

Response: Thank you for this important observation. The parameter sigma=0.001 was selected to enforce a stricter curvature condition, which helps preserve descent properties and improve numerical stability for the proposed hybrid CG method. We have added a justification of this choice in the numerical section and clarified its effect on convergence behavior.

Comment 4: The manuscript does not have published code, and lack implementation details.

Response: Thank you for the comment. Additional implementation details have been included in the numerical section, and a statement has been added indicating that the MATLAB implementation of the proposed method is available upon request to ensure reproducibility.

Comment 5: Inconsistency issues exist. For example, Algorithm 4.5 is mentioned twice but never defined.

Response: Thank you for the comment. The algorithm has already been defined in the manuscript. To avoid confusion, we have carefully revised the algorithm numbering and cross-references to ensure consistency throughout the manuscript..

<u>Reviewer 3</u>: This manuscript presents a new unconstrained optimization method, which the authors call TTMZHS. This new method is inspired by the TTZHS and the TTRMIL methods. The authors provide theoretical proofs for sufficient descent conditions and global convergence and they also evaluate its efficiency using 273 different benchmark functions as well as a real-world application in a LCSC model. In my opinion, the paper addresses a relevant topic, introduces a novel method, and is well written. However, there are some specific issues that need clarification:

Major comments:

Comment 1: In Theorem 2.1. and in Theorem 2.5. there are references to algorithm 4.5. However, I have not found this algorithm in the manuscript.

Response: Thank you for the comment. The algorithm has already been defined in the manuscript. To avoid confusion, we have carefully revised the algorithm numbering and cross-references to ensure consistency throughout the manuscript.

Comment 2: Why did the authors use sigma = 0.001 for the strong Wolfe conditions? Usually, much larger values are used.

Response: Thank you for this important observation. The parameter sigma=0.001 was selected to enforce a stricter curvature condition, which helps preserve descent properties and improve numerical stability for the proposed hybrid CG method. We have added a justification of this choice in the numerical section and clarified its effect on convergence behavior.

Comment 3: More details should be given regarding the failures on the different benchmark problems (e.g., TTMZHS failed on 43 out of 273, how many of these were due to the 2000 iterations limit?)

Response: Thank you for the comment. We have given the reasons why it failed.

Comment 4: In some of the profile curves, TTMZHS and TTZHS overlap or cross at low tau values. A clearer distinction or a table for small tau values would help interpret these figures more clearly and allow for a better comparison of the results between TTMZHS and TTZHS.

Response: Response: Thank you for the helpful suggestion. We have added additional clarification in the manuscript.

Response to Editor

We sincerely thank the Editor for the careful handling of our manuscript and for facilitating the review process. We also appreciate the valuable feedback provided, which has helped us improve the quality and clarity of our work. We have made every effort to address all comments and to correct any misprints identified during the revision process.

In the following, we present a detailed account of the revisions made, along with our responses to the comments received.

Comment 1: Please ensure that your manuscript meets PLOS ONE's style requirements, including those for file naming. The PLOS ONE style templates.

Response: Thank you for the reminder. We have carefully revised the manuscript to ensure that it complies with all PLOS ONE style requirements, including the file naming conventions and formatting guidelines.

Comment 2: Please note that PLOS One has specific guidelines on code sharing for submissions in which author-generated code underpins the findings in the manuscript. In these cases, we expect all author-generated code to be made available without restrictions upon publication of the work. Please review our guidelines.

Response: Thank you for the clarification. We confirm that all author-generated code underlying the findings of this manuscript will be made fully available without restriction upon publication, in accordance with PLOS ONE's guidelines.

Comment 3: Thank you for stating the following financial disclosure:

"This research is funded by Directorate of Research Funding and Ecosystem, Universitas Indonesia under Hibah PUTI 2025 (Grant No. PKS-356/UN2.RST/HKP.05.00/2025)."

Please state what role the funders took in the study. If the funders had no role, please state: "The funders had no role in study design, data collection and analysis, decision to publish, or preparation of the manuscript."

If this statement is not correct you must amend it as needed.

Please include this amended Role of Funder statement in your cover letter; we will change the online submission form on your behalf.

Response: Thank you for the note. We confirm that this statement has been included in the new cover letter.

Comment 4: Please note that funding information should not appear in any section or other areas of your manuscript. We will only publish funding information present in the Funding Statement section of the online submission form. Please remove any funding-related text from the manuscript.

Response: Thank you for the clarification. We confirm that all funding-related information has been removed from the manuscript and is now provided only in the Funding Statement section of the submission form.

Comment 5: We note that your Data Availability Statement is currently as follows:

"All relevant data are within the manuscript and its Supporting Information files."

Please confirm at this time whether or not your submission contains all raw data required to replicate the results of your study.

Response: Thank you for the inquiry. We confirm that there are no raw data associated with this study. All relevant information necessary to understand and replicate the results is already provided within the manuscript.

Comment 5: If the reviewer comments include a recommendation to cite specific previously

published works, please review and evaluate these publications to determine whether they are relevant and should be cited. There is no requirement to cite these works unless the editor has indicated otherwise.

Response: Thank you for the guidance. We have carefully reviewed the suggested references and evaluated their relevance. Only those works that are directly relevant to our study have been incorporated into the revised manuscript.
