# Supplementary material for: A three-term conjugate gradient method with a new descent structure for unconstrained optimization and low-carbon supply chain optimization
Source: PLoS One. 2026 Aug 27;21(8):e0356498. doi: 10.1371/journal.pone.0356498 (PMC13521400; doi:10.1371/journal.pone.0356498)
Supplement: Peer review history

# Peer review: Decision Letter 0

**DOI:** 10.1371/journal.pone.0356498.r001

**Authors:** Hoda Bidkhori

13 Feb 2026

Dear Dr. Malik,

Thank you for submitting your manuscript to PLOS ONE. After careful consideration, we feel that it has merit but does not fully meet PLOS ONE’s publication criteria as it currently stands. Therefore, we invite you to submit a revised version of the manuscript that addresses the points raised during the review process.

Two reviewers have recommended major revision and one reviewer has recommended minor revision for this manuscript. After carefully considering the reviewers’ evaluations and comments, I agree with their assessments.

Please submit your revised manuscript by Mar 30 2026 11:59PM. If you will need more time than this to complete your revisions, please reply to this message or contact the journal office at plosone@plos.org. When you're ready to submit your revision, log on to https://www.editorialmanager.com/pone/ and select the 'Submissions Needing Revision' folder to locate your manuscript file.

- A letter that responds to each point raised by the academic editor and reviewer(s). You should upload this letter as a separate file labeled 'Response to Reviewers'.
- A marked-up copy of your manuscript that highlights changes made to the original version. You should upload this as a separate file labeled 'Revised Manuscript with Track Changes'.
- An unmarked version of your revised paper without tracked changes. You should upload this as a separate file labeled 'Manuscript'.

If you would like to make changes to your financial disclosure, please include your updated statement in your cover letter. Guidelines for resubmitting your figure files are available below the reviewer comments at the end of this letter.

If applicable, we recommend that you deposit your laboratory protocols in protocols.io to enhance the reproducibility of your results. Protocols.io assigns your protocol its own identifier (DOI) so that it can be cited independently in the future. For instructions see: https://journals.plos.org/plosone/s/submission-guidelines#loc-laboratory-protocols. Additionally, PLOS ONE offers an option for publishing peer-reviewed Lab Protocol articles, which describe protocols hosted on protocols.io. Read more information on sharing protocols at https://plos.org/protocols?utm_medium=editorial-email&utm_source=authorletters&utm_campaign=protocols.

We look forward to receiving your revised manuscript.

Kind regards,

Hoda Bidkhori

Academic Editor

PLOS One

**Journal Requirements:**

When submitting your revision, we need you to address these additional requirements.

1. Please ensure that your manuscript meets PLOS ONE's style requirements, including those for file naming. The PLOS ONE style templates can be found at

https://journals.plos.org/plosone/s/file?id=wjVg/PLOSOne_formatting_sample_main_body.pdf and

https://journals.plos.org/plosone/s/file?id=ba62/PLOSOne_formatting_sample_title_authors_affiliations.pdf

2. Please note that PLOS One has specific guidelines on code sharing for submissions in which author-generated code underpins the findings in the manuscript. In these cases, we expect all author-generated code to be made available without restrictions upon publication of the work. Please review our guidelines at https://journals.plos.org/plosone/s/materials-and-software-sharing#loc-sharing-code and ensure that your code is shared in a way that follows best practice and facilitates reproducibility and reuse.

3. Thank you for stating the following financial disclosure:

“This research is funded by Directorate of Research Funding and Ecosystem, Universitas Indonesia under Hibah PUTI 2025 (Grant No. PKS-356/UN2.RST/HKP.05.00/2025).”

Please state what role the funders took in the study.  If the funders had no role, please state: "The funders had no role in study design, data collection and analysis, decision to publish, or preparation of the manuscript."

If this statement is not correct you must amend it as needed.

Please include this amended Role of Funder statement in your cover letter; we will change the online submission form on your behalf.

4. Please note that funding information should not appear in any section or other areas of your manuscript. We will only publish funding information present in the Funding Statement section of the online submission form. Please remove any funding-related text from the manuscript.

5. We note that your Data Availability Statement is currently as follows:

“All relevant data are within the manuscript and its Supporting Information files.”

Please confirm at this time whether or not your submission contains all raw data required to replicate the results of your study. Authors must share the “minimal data set” for their submission. PLOS defines the minimal data set to consist of the data required to replicate all study findings reported in the article, as well as related metadata and methods (https://journals.plos.org/plosone/s/data-availability#loc-minimal-data-set-definition).

For example, authors should submit the following data:

- The values behind the means, standard deviations and other measures reported;

- The values used to build graphs;

- The points extracted from images for analysis.

Authors do not need to submit their entire data set if only a portion of the data was used in the reported study.

If your submission does not contain these data, please either upload them as Supporting Information files or deposit them to a stable, public repository and provide us with the relevant URLs, DOIs, or accession numbers. For a list of recommended repositories, please see https://journals.plos.org/plosone/s/recommended-repositories.

If there are ethical or legal restrictions on sharing a de-identified data set, please explain them in detail (e.g., data contain potentially sensitive information, data are owned by a third-party organization, etc.) and who has imposed them (e.g., an ethics committee). Please also provide contact information for a data access committee, ethics committee, or other institutional body to which data requests may be sent. If data are owned by a third party, please indicate how others may request data access.

6. If the reviewer comments include a recommendation to cite specific previously published works, please review and evaluate these publications to determine whether they are relevant and should be cited. There is no requirement to cite these works unless the editor has indicated otherwise.

**Additional Editor Comments:**

This paper proposes a new three-term conjugate gradient method, called TTMZHS, for solving large-scale unconstrained optimization problems. The method is inspired by two existing three-term methods, TTZHS and TTRMIL, and modifies their parameter structure to improve computational stability and performance. The authors establish theoretical properties of the method, including sufficient descent and global convergence under the strong Wolfe line search conditions. Extensive numerical experiments on 273 benchmark problems demonstrate improved efficiency compared to related methods in terms of iterations, CPU time, function evaluations, and gradient evaluations.

In addition to theoretical and numerical validation, the method is applied to a Low Carbon Supply Chain (LCSC) optimization model. The application optimizes pricing, warranty period, and carbon emission reduction decisions under a carbon trading framework. The results show that TTMZHS outperforms comparison methods in this real-world setting, demonstrating both theoretical robustness and practical effectiveness.

Reviewer 1 suggested minor revision, while Reviewer 2 and Reviewer 3 suggested major revision.

I suggest proceeding with a major revision, addressing all reviewer comments, including the following:

Reviewer 1 Suggestions (Minor)

Improve language and grammar throughout the manuscript.

Clarify the motivation and design intuition behind combining TTZHS and TTRMIL in Section 2.1.

Add a smoother introductory paragraph before the LCSC application explaining the problem’s challenges and why CG-type methods are appropriate.

Reviewer 2 Suggestions (Major)

Strengthen the novelty justification by clearly distinguishing TTMZHS theoretically from TTZHS and TTRMIL.

Expand the literature review to include additional three-term CG developments and well-established nonlinear CG methods (e.g., Hager–Zhang and related follow-up work).

Extend numerical comparisons to include additional benchmark methods where appropriate.

Justify the choice of strong Wolfe parameters, particularly σ = 0.001, and include discussion on parameter sensitivity.

Provide more implementation details and clarify reproducibility aspects.

Correct the inconsistency regarding the undefined reference to “Algorithm 4.5.”

Reviewer 3 Suggestions (Major)

Define and clarify the missing “Algorithm 4.5.”

Justify the use of σ = 0.001 in the strong Wolfe conditions.

Provide detailed explanation of benchmark failures, including how many are due to the 2000-iteration limit.

Improve clarity of performance profile comparisons, especially where TTMZHS and TTZHS overlap at small τ value

[Note: HTML markup is below. Please do not edit.]

Reviewers' comments:

Reviewer's Responses to Questions

**Comments to the Author**

1. Is the manuscript technically sound, and do the data support the conclusions?

Reviewer #1: Yes

Reviewer #2: Yes

Reviewer #3: Yes

**********

2. Has the statistical analysis been performed appropriately and rigorously?

Reviewer #1: Yes

Reviewer #2: N/A

Reviewer #3: N/A

**********

3. Have the authors made all data underlying the findings in their manuscript fully available?

The PLOS Data policy

Reviewer #1: Yes

Reviewer #2: No

Reviewer #3: Yes

**********

4. Is the manuscript presented in an intelligible fashion and written in standard English?

Reviewer #1: No

Reviewer #2: Yes

Reviewer #3: Yes

**********

Reviewer #1: The manuscript presents a new hybrid three-term conjugate gradient method (TTMZHS) for solving unconstrained optimization problems. The authors establish sufficient descent and global convergence properties under the strong Wolfe line search and validate the method through comprehensive numerical experiments on 273 benchmark functions. The inclusion of a Low Carbon Supply Chain (LCSC) optimization example demonstrates the practical relevance and applicability of the proposed approach.

The manuscript offers a meaningful contribution to the field of numerical optimization and is supported by strong theoretical and computational results. I believe the paper is fundamentally sound and suitable for publication in PLOS ONE after minor revisions primarily concerning writing clarity and small improvements in exposition.

Minor Comments:

(i) Language and Grammar: Please carefully proofread the manuscript to correct grammatical errors and improve readability. For example, the Abstract sentence “In this paper proposes…” should be revised to “This paper proposes…”. Several similar issues appear throughout the text.

(ii) Motivation for the Hybrid Method: In Section 2.1, briefly clarify why TTZHS and TTRMIL were chosen to be combined. A short explanation of the design intuition behind TTMZHS would help readers follow the logic.

(iii) Low Carbon Supply Chain Application: The transition to the LCSC model is abrupt. Please add a brief introductory paragraph explaining why this problem is challenging and why CG-type methods are appropriate.

Reviewer #2: Summary:

The manuscript proposed a modified three-term conjugate gradient (CG) method for unconstrained smooth optimization, named TTMZHS. The new method focuses on the denominators of two scalars. Comprehensive analysis was conducted to support the theoretical properties of convergence, and showed empirical superior over two other three term methods. A case study applying the method to a low-carbon supply chain profit optimization model that shows superior performance.

Strength:

1. Clear model definition.

2. Comprehensive convergence analysis.

3. Comprehensive analysis over a large benchmark set.

4. Application is aligned with the scope of Plos One.

Weakness:

1. Novelty is limited, as the paper combines components from prior TTZHS and TTRMIL directions. Beyond performance, theoretical justification would be help to distinguish from existing methods.

2. Insufficient literature review with limited benchmarks. Only two close relatives (TTZHS and TTRMIL) are compared.

CG is widely studied and used in the literature. For example, there is a follow up paper of TTRMIL published in 2020 “Some three-term conjugate gradient methods with the new direction structure.” Well-established nonlinear CG like Hager–Zhang is also omitted in the literature review and experiment, while its test function is included in the experiment.

A more comprehensive review is needed as well.

3. The numerical section states strong Wolfe constants psi=10^-4 and sigma=0.001. However, in typical strong Wolfe implementations, sigma is much larger (also see the 2020 paper mentioned above). Justification of this choice, and an additional sensitivity analysis is needed to justify the choice of parameters.

4. The manuscript does not have published code, and lack implementation details.

5. Inconsistency issues exist. For example, Algorithm 4.5 is mentioned twice but never defined.

Reviewer #3: This manuscript presents a new unconstrained optimization method, which the authors call TTMZHS. This new method is inspired by the TTZHS and the TTRMIL methods. The authors provide theoretical proofs for sufficient descent conditions and global convergence and they also evaluate its efficiency using 273 different benchmark functions as well as a real-world application in a LCSC model. In my opinion, the paper addresses a relevant topic, introduces a novel method, and is well written. However, there are some specific issues that need clarification:

- In Theorem 2.1. and in Theorem 2.5. there are references to algorithm 4.5. However, I have not found this algorithm in the manuscript.

- Why did the authors use sigma = 0.001 for the strong Wolfe conditions? Usually, much larger values are used.

- More details should be given regarding the failures on the different benchmark problems (e.g., TTMZHS failed on 43 out of 273, how many of these were due to the 2000 iterations limit?)

- In some of the profile curves, TTMZHS and TTZHS overlap or cross at low tau values. A clearer distinction or a table for small tau values would help interpret these figures more clearly and allow for a better comparison of the results between TTMZHS and TTZHS.

**********

what does this mean?). If published, this will include your full peer review and any attached files.

If you choose “no”, your identity will remain anonymous but your review may still be made public.

**Do you want your identity to be public for this peer review?**  For information about this choice, including consent withdrawal, please see our Privacy Policy

Reviewer #1: No

Reviewer #2: No

Reviewer #3: **Yes:** David Hervás Marín

**********

[NOTE: If reviewer comments were submitted as an attachment file, they will be attached to this email and accessible via the submission site. Please log into your account, locate the manuscript record, and check for the action link "View Attachments". If this link does not appear, there are no attachment files.]

To ensure your figures meet our technical requirements, please review our figure guidelines: https://journals.plos.org/plosone/s/figures

You may also use PLOS’s free figure tool, NAAS, to help you prepare publication quality figures: https://journals.plos.org/plosone/s/figures#loc-tools-for-figure-preparation.

NAAS will assess whether your figures meet our technical requirements by comparing each figure against our figure specifications.

# Author comment: Author response to Decision Letter 1

**DOI:** 10.1371/journal.pone.0356498.r002

6 Apr 2026

We thank the reviewer and editor for his/her careful reading, and for raising relevant concerns. We tried our best to correct all the misprints we observe during the revision. We have attached a response file to the comments provided and a manuscript file that has been revised according to the comments.

We hope that my article can be accepted and published quickly.

Attachment Submitted filename: Response to Reviewers (2).pdf

# Peer review: Decision Letter 1

**DOI:** 10.1371/journal.pone.0356498.r003

**Authors:** Prabhujit Mohapatra

5 Aug 2026

A Three-Term Conjugate Gradient Method with a New Descent Structure for Unconstrained Optimization and Low-Carbon Supply Chain Optimization

PONE-D-25-55566R1

Dear Dr. Malik,

We’re pleased to inform you that your manuscript has been judged scientifically suitable for publication and will be formally accepted for publication once it meets all outstanding technical requirements.

Within one week, you’ll receive an e-mail detailing the required amendments. When these have been addressed, you’ll receive a formal acceptance letter and your manuscript will be scheduled for publication.

An invoice will be generated when your article is formally accepted. Please note, if your institution has a publishing partnership with PLOS and your article meets the relevant criteria, all or part of your publication costs will be covered. Please make sure your user information is up-to-date by logging into Editorial Manager at Editorial Manager® and clicking the ‘Update My Information' link at the top of the page. For questions related to billing, please contact billing support.

If your institution or institutions have a press office, please notify them about your upcoming paper to help maximize its impact. If they’ll be preparing press materials, please inform our press team as soon as possible -- no later than 48 hours after receiving the formal acceptance. Your manuscript will remain under strict press embargo until 2 pm Eastern Time on the date of publication. For more information, please contact onepress@plos.org.

Kind regards,

Prabhujit Mohapatra, PhD

Academic Editor

PLOS One

Additional Editor Comments (optional):

Reviewers' comments:

Reviewer's Responses to Questions

**Comments to the Author**

Reviewer #1: All comments have been addressed

Reviewer #2: All comments have been addressed

**********

2. Is the manuscript technically sound, and do the data support the conclusions?

Reviewer #1: Yes

Reviewer #2: Yes

**********

3. Has the statistical analysis been performed appropriately and rigorously?

Reviewer #1: Yes

Reviewer #2: N/A

**********

4. Have the authors made all data underlying the findings in their manuscript fully available?

The PLOS Data policy

Reviewer #1: No

Reviewer #2: No

**********

5. Is the manuscript presented in an intelligible fashion and written in standard English?

Reviewer #1: Yes

Reviewer #2: Yes

**********

Reviewer #1: Thank you for carefully addressing all of my comments in full. I am pleased with the revised version of the manuscript and am happy to accept it for publication.

Reviewer #2: I would like to thank the authors for taking their time to address the comments in detail. Other than the code is not currently available and subject to request, I do not have any further questions. I look forward to trying the codes during the proof read process where codes shall be included in the manuscript at that time.

**********

what does this mean?). If published, this will include your full peer review and any attached files.

If you choose “no”, your identity will remain anonymous but your review may still be made public.

**Do you want your identity to be public for this peer review?**  For information about this choice, including consent withdrawal, please see our Privacy Policy

Reviewer #1: No

Reviewer #2: No

**********

# Editor report: Acceptance letter

**DOI:** 10.1371/journal.pone.0356498.r004

**Authors:** Prabhujit Mohapatra

PONE-D-25-55566R1

PLOS One

Dear Dr. Malik,

I'm pleased to inform you that your manuscript has been deemed suitable for publication in PLOS One. Congratulations! Your manuscript is now being handed over to our production team.

At this stage, our production department will prepare your paper for publication. This includes ensuring the following:

* All references, tables, and figures are properly cited

* All relevant supporting information is included in the manuscript submission,

* There are no issues that prevent the paper from being properly typeset

You will receive further instructions from the production team, including instructions on how to review your proof when it is ready. Please keep in mind that we are working through a large volume of accepted articles, so please give us a few days to review your paper and let you know the next and final steps.

Lastly, if your institution or institutions have a press office, please let them know about your upcoming paper now to help maximize its impact. If they'll be preparing press materials, please inform our press team within the next 48 hours. Your manuscript will remain under strict press embargo until 2 pm Eastern Time on the date of publication. For more information, please contact onepress@plos.org.

You will receive an invoice from PLOS for your publication fee after your manuscript has reached the completed accept phase. If you receive an email requesting payment before acceptance or for any other service, this may be a phishing scheme. Learn how to identify phishing emails and protect your accounts at https://explore.plos.org/phishing.

If we can help with anything else, please email us at customercare@plos.org.

Thank you for submitting your work to PLOS One and supporting open access.

Kind regards,

PLOS ONE Editorial Office Staff

on behalf of

Dr. Prabhujit Mohapatra

Academic Editor

PLOS One
